# DOES - A multimodal dataset for supervised and unsupervised analysis of steel scrap

**DOI:** 10.1038/s41597-023-02662-6

**Published:** 2023-11-08

**Authors:** Michael Schäfer, Ulrike Faltings, Björn Glaser

**Affiliations:** 1 KTH Royal Institute of Technology, Department of Materials Science and Engineering, Stockholm, 10044 Sweden; 2 SHS - Stahl-Holding-Saar GmbH & Co. KGaA, Digitalization & AI, Dillingen, 66763 Germany

**Keywords:** Research data, Industry

## Abstract

*DOES* - Dataset of European scrap classes. Today, scrap is already an important raw material for industry. Due to the transformation to green steel, the secondary raw material scrap will become increasingly important in the coming years. With *DOES* a free dataset is presented, which represents common non-alloyed European scrap classes. Two important points were considered in this dataset. First, scrap oxidizes under normal external conditions and the visual appearance changes, which plays an important role in visual inspections. Therefore, *DOES* includes scrap images of different degrees of corrosion attack. Second, images of scrap metal (mostly scrap piles) usually have no intrinsic order. For this reason, a technique to extract many overlapping rectangles from raw images was used, which can be used to train deep learning algorithms without any disadvantage. This dataset is very suitable to develop industrial applications or to research classification algorithms. The dataset was validated by experts and through machine learning models.

## Background & Summary

On the way to climate-neutral production in the steel industry, a reduction of *CO*_2_ emissions of 80–95% could be achieved by 2050 compared to 1990^1^. To achieve this goal, the steel industry is currently facing major challenges to significantly reduce its direct and indirect *CO*_2_ emissions. Besides the switch to hydrogen-based production and migration to the EAF (Electronic Arc Furnace) production route, the use and recycling of steel scrap and the development of new technologies to Technology Readiness Level 8 is a key factor^1^. As a result, the demand for high-quality scrap in the steel industry is increasing, but the availability of this secondary raw material will decrease in the future. However, the amount of old scrap that can be used is expected to increase^2^. This induces high demands and requires new strategies for scrap cleaning, scrap sorting, scrap processing and scrap disposal^3^. Digitalization and machine learning are fundamental tools for implementing these strategies and developing new systems, products and processes^4^. Structured, semi-structured and unstructured data form the basis for the implementation of these digital technologies. It is becoming increasingly important for companies to collect, store and process relevant data. In order to successfully implement digitalization and the green transformation of the steel sector, demand for data, tools and intelligent applications will increase significantly. *DOES* can help to develop intelligent scrap yard, scrap inspection and classification systems.

In contrast to datasets such as MS COCO^5^ or ImageNet^6^ featuring images for “object or thing” categories^7,8^, *DOES* focuses on specific “stuff”-like categories. Thing classes or categories have specific features, specific sizes, particular shapes or attributes that belong to this object (e.g. a cat has ears, legs and eyes). In contrast, stuff classes do not have such special properties. For everyday object categories such as cars or people, a vast number of datasets have been collected and annotated and much research has been conducted on image categorization, instance detection, semantic segmentation, instance segmentation, etc. However, for tasks such as scrap classification or more generally “stuff” classification, research and dataset collection have been far more limited as yet, see e.g.^9,10^. There are a few datasets for surface materials or general “stuff” categories such as COCO-Stuff^10^, CUReT^11^, Flickr Materials Database (FMD)^12^, KTH-TIPS^13,14^, OpenSurfaces^15^ or Materials in Context (MINC)^16^, but these focus on classifying different materials categories, e.g. wood vs. metal.

To the best of the authors’ knowledge, *DOES* is the first freely available steel scrap dataset, covering the defined non-alloyed European steel scrap grades (Table 1). There are some commercially available solutions for automatized scrap discrimination (e.g. www.primetals.com, www.automation-fair.com) as well as some non-public internal solutions using visual characteristics developed at scrap processing companies such as steel manufacturers. But there are no independently evaluated systems providing a baseline against which *DOES* could be validated. Many publications and research activities on scrap classification focus on shredded scrap, non-ferrous materials and techniques such as LIBS (laser induced breakdown spectroscopy) or multi-spectral image analysis, e.g.^17–23^. Publications focusing on ferrous scrap classification in steel plant settings do not provide reference to the dataset used or discuss classification techniques without providing classification results, e.g.^9,24–26^. In many steel plants, classification is done mostly by manual visual inspection.

**Table 1:** Different scrap classes and their descriptions & dimensions.

Category	Scrap ID	Description	Dimension
used scrap	E1	Old thin steel scrap	≤1.5 × 0.5 × 0.5 m Thickness <6 mm
	E3	Old thick steel scrap	≤1.5 × 0.5 × 0.5 m Thickness ≥6 mm
new scrap	E2	Thick new production steel scrap	<1,5 × 0,5 × 0,5 m Thickness ≥3 mm
	E6	Thin new production steel scrap (compressed or firmly baled)	Thickness <3 mm
	E8	Thin new production steel scrap	≤1.5 × 0.5 × 0.5 m Thickness <3 mm
steel turnings	E5H	Homogeneous lots of carbon steel turnings	—
high residual scrap	EHRB	Old and new steel scrap consisting mainly of rebars and merchant bars	max 1,5 × 0,5 × 0,5 m
shredded	E40	Shredded steel scrap	—
background	—	Different background images	—

This scrap dataset can provide a basis for developing automatized scrap classification systems using computer vision approaches or other scrap-related solutions. Freely available datasets are very useful for many purposes and stakeholders, for example application developers using it for testing and training machine learning models, educational purposes to provide a reference of the scope in scrap classes, or research on scrap discrimination. Moreover, *DOES* can also be of interest to researchers, data scientists, students and for general deep learning purposes, providing an alternative to the “classical” object-category datasets. In some sense scrap is an interesting fusion of object-like characteristics (richness of features) with stuff-characteristics (translation-, rotation-, orientation-, section-invariance as to detectability of class characteristics):

Scrap tends to be very heterogeneous in terms of item-sizes or shapes. Moreover, the scrap images intrinsically do not have a dedicated orientation or structure. The latter means the presence of certain characteristics in one section of the image does not reliably generate an implication on to-be-expected characteristics for other sections of the image (in contrast to “object” categories, where e.g. a cat’s nose in one part of the image makes it a fair guess that the cat’s eyes will be located above to the left and right with regard to the nose’s orientation). So, the scrap characteristics are a local property, not a global one.

**Fig. 1:**
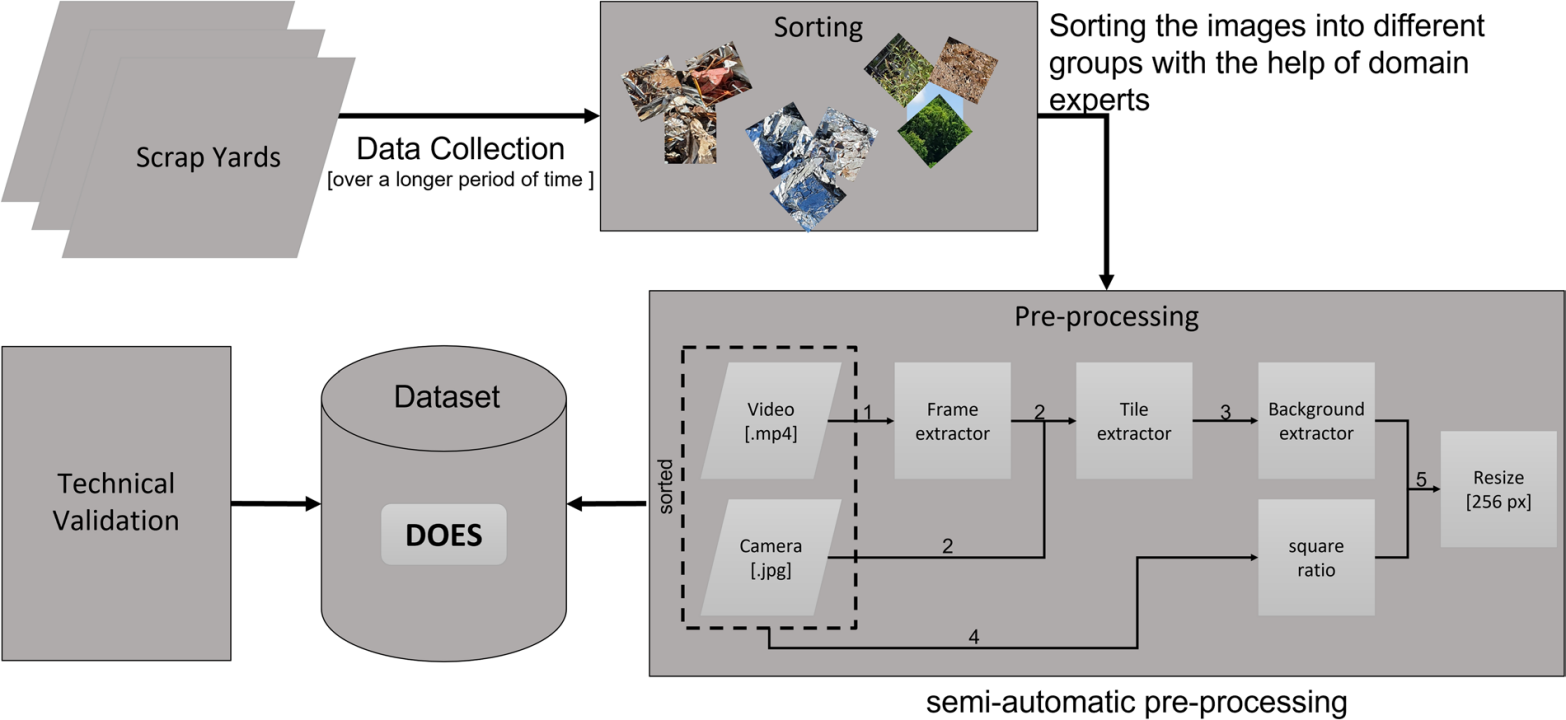
Overview of the approach and methodology.

The fact that a local perspective offers a similar richness of features as a global perspective and is sufficient to discern the characteristics of the scrap in the given section is used to generate a larger dataset with moderate effort by extracting multiple tiles from a given camera-shot and using these as individual dataset instances, rather than scaling down standard camera images to a size suitable for training. In particular for image classification task annotations, this can also decrease the annotation effort, and ensures a fixed input size for classification models. Moreover, as some tiles will depict the foreground and others the background, this also encourages robustness to scale variation. The size of the tiles was chosen to be a useful input size for Convolutional Networks while staying in a range where scrap characteristics are well discernible, thus maximizing the uptake of dataset instances from a single camera shot. This technique is not limited to scrap images, but could be applied to any dataset where to-be-discerned characteristics are a local property and do not rely on the global perspective. When creating the dataset, several steps were performed. An overview of the approach and the process is shown in Fig. 1, which is divided into the following main steps: (I) Image and Video collection; (II) Manually sort the images and videos into different classes; (III) Pre-processing of the sorted recordings; (IV) Sorting into the *DOES* dataset; (V) Technical validation. Since the dataset was created over several months, most of the various process steps were performed several times. Validation with machine learning methods were performed only at the end on the final dataset.

In summary, *DOES* provides a new opportunity for researchers to investigate computer vision challenges aside from the classical “object”-centered topics. A broader understanding and research on the mechanisms and workings of CV approaches on different types of datasets can hopefully help the field of research in general. It could be an interesting path for further research to investigate structural differences as well as things in common between “object”- and “stuff”-related tasks further as well as ways in which models can benefit from both worlds. The experiments conducted for the validation of the model show the usability of the dataset and the structural difference from common datasets.

The construction of *DOES* is described in more detail in section Methods. In particular, the composition and collection is described in subsection Dataset, the pre-processing in subsection Pre-processing, the basic structure in section Data Records and the validation of *DOES* in section Technical Validation.

## Methods

In this section, an explanation of the general structure and steps for collecting and pre-processing of the dataset is given.

### Dataset

The scrap images were collected at the scrapyards of Dillinger, Saarstahl AG, affiliated entities of SHS (SHS-Stahl-Holding-Saar GmbH & Co. KGaA https://www.stahl-holding-saar.de/shs/en/home/index.shtml is an operational management holding company that actively performs tasks for the two major steel companies in Saarland, Aktien-Gesellschaft der Dillinger Hüttenwerke (Dillinger) https://www.dillinger.de/d/en/corporate/index.shtml and Saarstahl https://www.saarstahl.com/sag/en/products/index.shtml) and local transshipment scrap yards. *DOES* basically covers the defined European steel scrap grades and an additional background category (Table 1). These scrap types also define the hierarchical structure of the dataset.

**Fig. 2:**
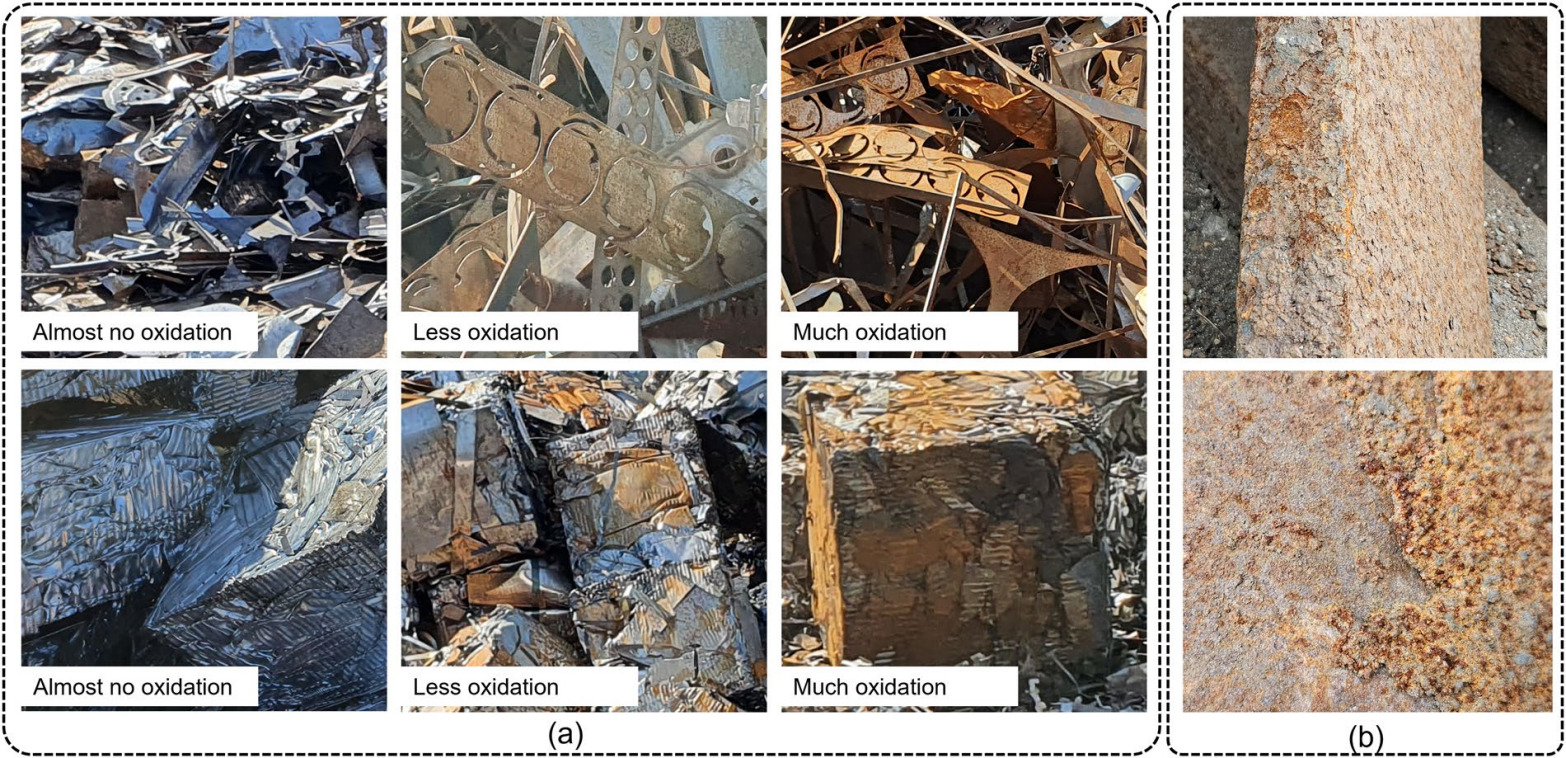
(**a**) A typical surface change over the time in the case of oxidation (**b**) Heavy friable and flaky surface change.

The images were collected over several months at different times of the day and under different weather conditions. This is especially important in order to be able to map the different states of the scrap surface (Fig. 2a); due to the fact that when oxygen and water act on the iron and steel, this oxidation reaction forms hydrated ferric oxide (*Fe*_2_*O*_3_), i.e. rust. Surface rust is usually friable and flaky and takes different colors (Fig. 2b).

This has a similar effect on classification using deep learning as Suharjito *et al*.^27^ have described for oil palm fruits. Often Deep Learning systems are trained with grayscale images. But color can play an important role in a visual inspection of scrap, whether manually or using machine learning. This is because different grades or elements can also be distinguished by color or surface. Good examples are copper, alloying elements such as zinc or various stainless steels.

To ensure that the test images are definitely different from the original dataset, the collection of images for the test set was made temporally independent of the main dataset. This also allowed to ensure that no scenes from the train set were duplicated when creating the test set rectangles.

Scrap is most often stored in piles or containers prior to use or further processing. For that reason, different recording techniques are required for the images in order to be able to represent the viewing angle of a camera on a loading crane, for example. Therefore, various smartphone cameras and a drone were used to obtain image data. The collection of images in the dataset was made with different settings. Explicit care was taken to capture images with different angles, lighting conditions, distances, contrasts, colors, focal length, environments (indoor and outdoor), shadows, etc. This variance offers several benefits for future applications. For example, overfitting to a particular setting can be avoided through this diversity. As a result, inference can become much more robust in future applications with different camera technology and in different environments.

Images from the smartphone cameras used have a resolution of 4032 × 3024, 4624 × 3468 and 4000 × 3000 pixels respectively, whereas drone videos were recorded with a Full HD resolution of 1920 × 1080 pixels and a frame rate of 30 frames per second (fps). The raw images in 4:3 or 16:9 format were each cropped into two images with a square ratio. Here the side length of the square is the pixel size of the smaller side (see section Pre-processing). All images were resized to 256 × 256 pixels afterwards.

When scrap is used or further processed, scrap is usually stored in larger quantities. The displayed scrap on the raw images has no order or represents objects of its own. In contrast to other datasets, the spatial orientation of the scrap does not play a role in the subsequent inference or classification using machine learning methods. This allowed to divide the raw images into many overlapping tiles (see section Pre-processing) in a structured way. Thus, the variety of tiles allow efficient training. Tiles offer great advantage for this kind of unstructured data.

### Pre-processing

**Fig. 3:**
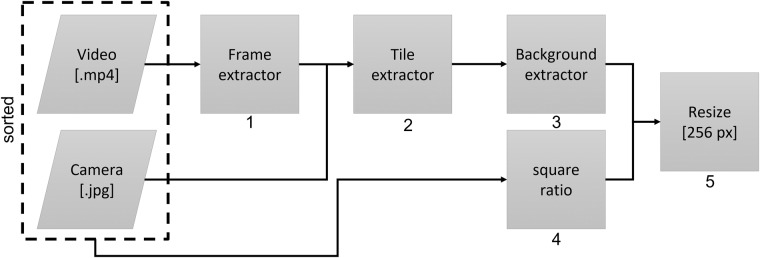
Pre-processing.

Only single images and no videos were recorded with the smartphone cameras. With the drone, only video recordings were made. This data was saved sorted by scrap categories as images (.png) resp. videos (.mp4). To create the dataset, five pre-processing steps (Fig. 3) were performed. The dataset should be easily and quickly expandable in the future. The manual sorting, parameterization, defining the tile size and step size is very time consuming. The creation of the dataset was semi-automated using various self-developed or customized Python programs.

#### Extracting frames from the videos

To exclude similar or almost redundant frames, it is necessary to extract frames from the videos that are sufficiently different from the previous frame. Since the drone videos were recorded at approximately constant speed, frames can be selected equidistantly. The videos are between 1 minute and 11 seconds and 1 minute and 49 seconds long. From each second of the individual videos (30 fps), 5 frames were saved with a Python program. In the case of 5 frames per second, every 6*th* frame had to be extracted. When recording the videos, the flight direction or the flight altitude was changed at various points. With these changes in direction, it can happen that the drone briefly remains in a very similar place. Then a lot of pictures are taken at one spot. For example, if the drone stops at one point for 2 seconds, 60 frames are saved. In this example one would have 10 almost identical images. Therefore, too similar images were picked out manually after extracting the frames and deleted from the dataset.

#### Choosing the tile size

**Fig. 4:**
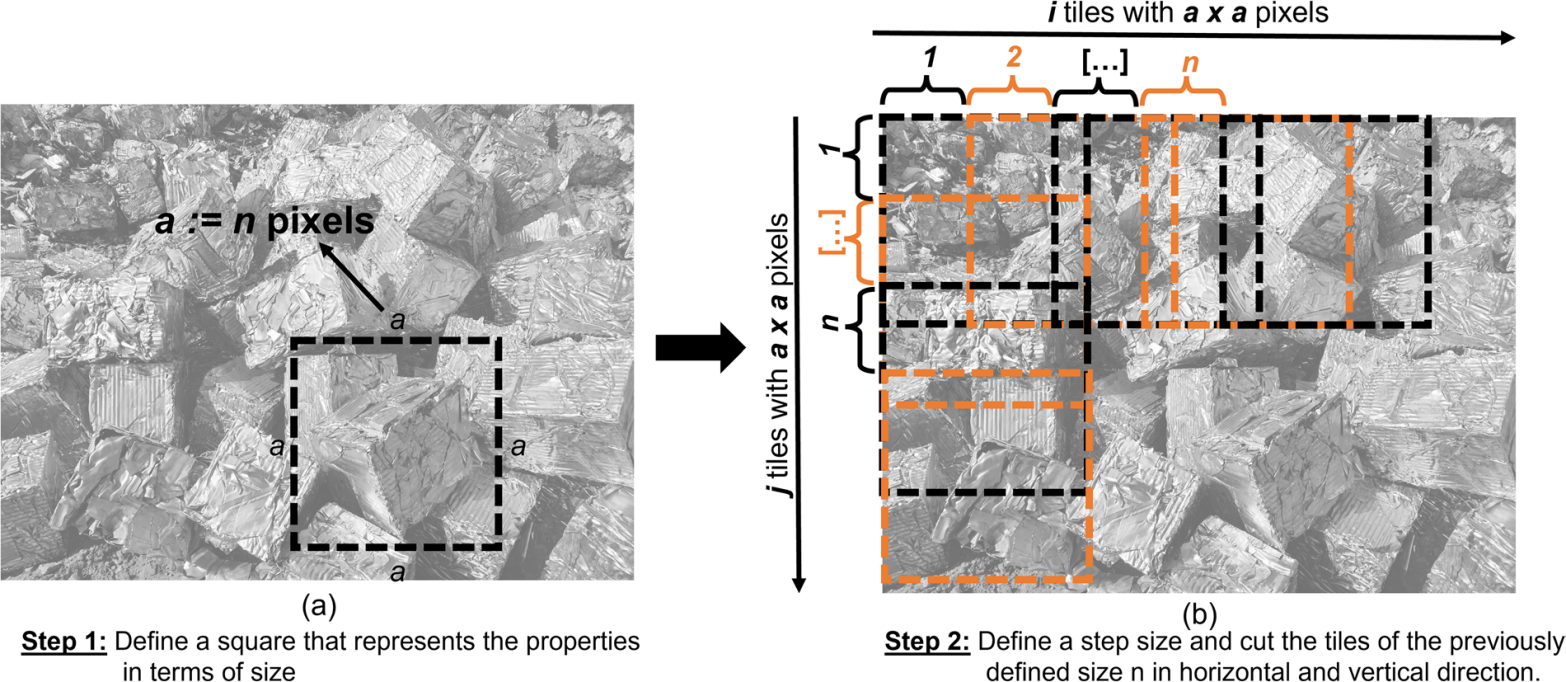
(**a**) Define the right size of a tile (**b**) Crop tiles with in (**a**) defined size.

A tile is defined as a square. However, the different scrap classes vary greatly in geometry and size. Therefore, the tile size was chosen so that the geometric properties of the scrap are still clearly visible (Fig. 4). The size of the tile was defined with the domain experts of the scrap warehouse and consumers in the steel plant. The defined tile size is as small as possible and as large as necessary. To avoid statistical artifacts by treating different classes differently, providing an implicit labeling for the different classes in the tiles, a fixed tile size was chosen for all the classes, not an individual size per class. As all tiles are finally being rescaled to a fixed pixel size, different initial tile sizes would result in different statistical artifacts from rescaling in the final dataset.

To choose the tile size, an experiment was conducted on a small subset of the raw images, containing smaller and larger examples from all the different classes and including views from different ranges. For each image, different sized tiles were extracted and it was validated through domain experts which tile size is large enough to still discern the scrap class characteristics.

The results of the experiment are related in Table 2. As shown in Table 2, only the classes E5H and E40 were clearly discernible in the smallest tile size of 256 px tested in the experiment. This is well understandable as both classes consist of very small scrap particles, as related in Table 1. For a tile size of 320 px, E1 and E6 were discernible as well, and for a tile size of 480 px, E2 was discernible in addition. Finally, for a tile size of 720 px, all the scrap classes were discernible on the tiles tested in the experiment. Based on the findings from the experiment, it was decided to use a tile-size of 720 px, as this was the smallest size where all classes were discernible.

**Table 2:** Discernibility of scrap class characteristics on extracted tiles depending on the tile size (x $\hat{=}$ discernible, - $\hat{=}$ non-discernible).

Tile size	E1	E2	E3	E5H	E6	E8	E40	EHRB
256	—	—	—	x	—	—	x	—
320	x	—	—	x	x	—	x	—
480	x	x	—	x	x	—	x	—
720	x	x	x	x	x	x	x	x
1024	x	x	x	x	x	x	x	x

#### Choosing the overlap for extraction of tiles

When extracting the tiles from the image, a certain overlap between adjacent tiles of between $\frac{1}{3}$ and $\frac{1}{2}$ of the tile size was allowed for. The overlap was chosen fixed for all cameras, but such that not to large a section of the initial image gets lost in the process.

The same overlap in both vertical and horizontal direction was chosen as tile extraction should not depend on whether the image is oriented with *width > height* or *height > width*. To choose the overlap, the larger of the two side-lengths of the images was regarded. Of course one could also have regarded both image dimensions, but as most raw images are oriented with *width > height* and the upper and lower section of the image are more likely to contain background (e.g. sky, soil, etc.), it was decided rather to go only by the larger of the two dimensions. The goal was to extract as many tiles as possible per image whilst remaining in the desired overlap range. Given an image resolution of *a* × *b*, with *a* > *b*, and a final tile size of *c*, and let *n* ∈ ℕ be the number of tiles to be extracted in a row from the length *a* with an overlap of size $o\in \left[\frac{1}{3}c,\frac{1}{2}c\right]\subset {\mathbb{R}}$. Then $a=c+(n-1)\cdot (c-o)\leftrightarrow c-\frac{a-c}{n-1}=o.$

So *n* needs to be chosen such that the overlap *o* lies in the desired range $\left[\frac{1}{3}720,\frac{1}{2}720\right]=\left[240,360\right]$ of pixels. The results for the camera resolutions used in the dataset collection are given in Table 3, rounded to two decimal points where necessary. Table 3 contains the values of *n* per image resolution where *o* lies inside the desired range [240, 360] as well as the largest *n* for which *o* < 240 and the smallest *n* for which *o* > 360, again per image resolution. In column **n** of Table 3, the largest value of *n* per image resolution value such that the overlap lies in the desired range is marked in boldface. To ensure a fixed overlap for all images, the mean of the overlaps was taken for the boldfaced *n*, rounded to an integer. Thus finally, an overlap of $339=\lfloor \frac{352+329.6+355.\bar{5}+320}{4}\rfloor$ px was chosen.

**Table 3:** Overlap *o* in pixel for the given resolutions given extracting *n* tiles per row in the larger image resolution value’s direction.

Larger image resolution value	n	o
4032	7	168
4032	8	246.89
4032	9	306
4032	**10**	352
4032	11	388.8
4624	9	232
4624	10	$286.\overline{2}$
4624	**11**	329.6
4624	12	$365.\overline{09}$
4000	7	$173.\overline{3}$
4000	8	251.43
4000	9	310
4000	**10**	$355.\overline{5}$
4000	11	392
1920	3	120
1920	**4**	320
1920	5	420

#### Sorting out backgrounds, square ratio and resize

In the raw images there are also areas that do not show scrap. These areas were defined as backgrounds in their own class and were sorted out manually from the image tiles. In a later step, a neural network could also take over the task and significantly increase the performance.

To be able to scale the raw images into a square format of 256 × 256 px, the raw images were also divided into squares. Due to the input images’ rectangular format with *shorter side* < *longer side* < 2 · *shorter side*, this could be achieved by dividing the images along the longer side into two squares with a slight overlap. These squared images were then resized to the desired format of 256 × 256 px.

## Data Records

The *DOES* is available at Zenodo (10.5281/zenodo.8219163^28^).

**Fig. 5:**
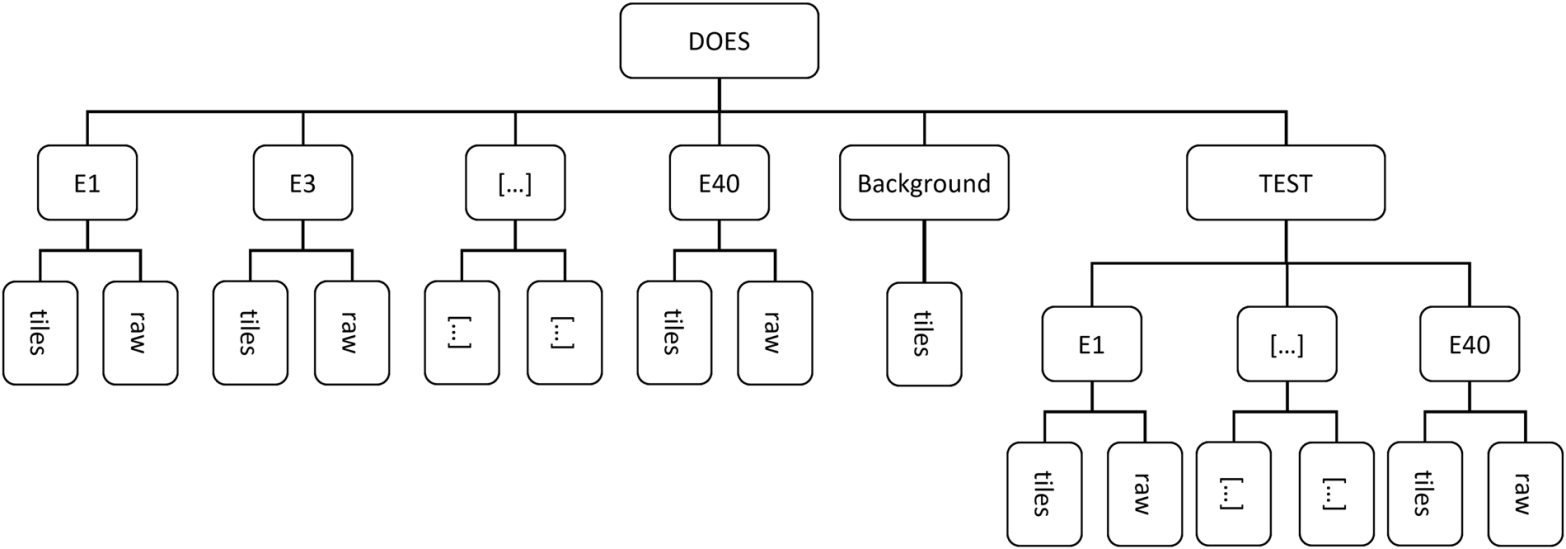
Schematic overview of the hierarchical structure of the dataset.

Figure 5 shows the hierarchical directory structure of the dataset. The root directory contains all other the directories of the individual classes. “Classes” is again divided into raw images and tile images. Due to this structure it is easily possible to create new individual training data with the required/desired classes. This structure already contains an implicit weak annotation of the different scrap classes. The basis of the dataset are the raw images in the raw directory, as the images in the tile directory were extracted from these images. There are no raw images where only the background is shown. The background directory contains only tiles extracted from images of all other classes. Additionally, there are images of the different classes in the test directory. These are images from indoor locations and outdoor locations pre-processed in the same way as the train set and are ideal for testing the future algorithms.

The statistics of *DOES* are given in Table 4. It can be seen from the different number of raw images and tiles that the classes are not equally balanced in the dataset. This is due to some classes being far more commonly used in (high quality) steel production than others, and some classes being far more heterogeneous than others; e.g. E5H, E40 have a comparatively homogeneous appearance whereas E1 can be very divers. As the primary sources of the images where Saarstahl’s and Dillinger’s scrapyards as well as suppliers’ scrapyards, the distribution of scrap in the dataset reflects the available scrap at these sites, which in return reflects the demand of a (high quality) steel production meltshop. Some lower quality scrap was included from local transshipment scrap yards, but these did not offer the full range of all scrap categories. The ratios of tiled images per class as compared to raw images of the same class in the train and test set vary between the different classes. This is due to several reasons: For one, some raw images have a lower resolution than others, thus resulting in less tiles per raw image than for other higher-resolution raw images. Another reason is that depending on the placement of the scrap on the scrap yard and the shape of the scrap piles, some raw images have a higher ratio of background as compared to scrap than others, thus resulting in more “background”-category tiles and less “scrap”-category tiles.

**Table 4:** *DOES* statistics: Number of raw image instances and tiles per class and in total, both for the train set and the test set (BG - Background).

Class	Train Set		Test Set	
	No. of raw instances	No. of tiles	No. of raw instances	No. of tiles
BG	0	1951	0	0
E1	1026	18236	36	1628
E2	232	10175	14	642
E3	1358	21254	38	1833
E5H	16	856	14	717
E6	1170	8841	26	1069
E8	1466	39496	20	927
E40	6	317	14	680
EHRB	746	4397	14	635
Total	6020	105523	176	8131

## Technical Validation

### Quality control

To rule out confusion and processing errors, the complete dataset was finally checked in detail by the domain experts and the authors. To ensure the accuracy and quality of the data set, images were checked in regular cycles. For this purpose, batches of images were manually controlled in different groups.

### Evaluation of the dataset

Several experiments were conducted to evaluate the dataset and the tile-approach. As framework, PyTorch was used. PyTorch is well suited for researchers^29^ to effectively develop convolutional neural networks for image classification. To technically validate the dataset, different variants of neural networks were trained on *DOES*. An overview and description of the experiments is provided in Table 5.

**Table 5:** Conducted experiments.

Experiment	Model No.	Architecture	Epochs	Batch Size	Class Weights	Train Set	Test Set
I	1	PreActResNet18^30^	50	32	yes	tiles	tiles
II	2	PreActResNet18	50	32	yes	raw	tiles
III	2	PreActResNet18	50	32	yes	raw	raw
IV	3	PreActResNet18	50	32	no	tiles	tiles
V	4	ResNet50^33^	50	32	no	tiles	tiles

During training, the best model was kept, i.e. not necessarily the model from epoch 50. The batch size was kept constant over all the experiments. Due to the imbalances in class sizes as visible in Table 4, class weights were used in experiments I,II,III. They were not used in experiments IV, V in account of the ResNet50 in experiment V being pretrained on a different dataset with other classes. Model 2 trained on the raw images was evaluated both on the tiled test set (experiment II) and the raw test set (experiment III). All other models 1, 3, 4 were trained on the tiled train set and only evaluated on the tiled test set (experiments I, IV, V). A more detailed motivation for the setup of the individual experiments is given in the following subsections.

An overview of the performance for the different experiments is provided in Table 6.

**Table 6:** Overall accuracy and per-Class accuracy on test set.

Experiment	Overall Accuracy	E1	E2	E3	E40	E5H	E6	E8	EHRB
I	**68.09**	**0.58**	0.32	**0.89**	**0.49**	0.48	**0.82**	0.87	**0.62**
II	28.93	0.09	0.07	0.52	0.00	0.01	0.33	0.62	0.42
III	43.18	0.19	0.21	0.66	0.14	0.07	0.65	**0.90**	0.21
IV	64.57	0.47	**0.33**	0.86	0.39	**0.60**	0.78	0.84	0.60
V	59.20	0.40	0.23	0.86	0.46	0.37	0.69	0.80	**0.62**

The evaluation of the model 2 on the raw test set (experiment III) is not as representative as for the tiled test set in experiment II since the size of the raw test dataset is quite small. For example, the very high per-class accuracy on E8 could also be a statistical artifact. Generally, there is quite a spread in performance in Table 6 between the different models. However, overall accuracy ranking is mostly consistent with per-class accuracy ranking, which is encouraging as it suggests that the learned features have a certain stability in being useful for class discrimination over the different classes.

The individual results are presented and discussed in more detail in the following.

#### Baseline model

**Fig. 6:**
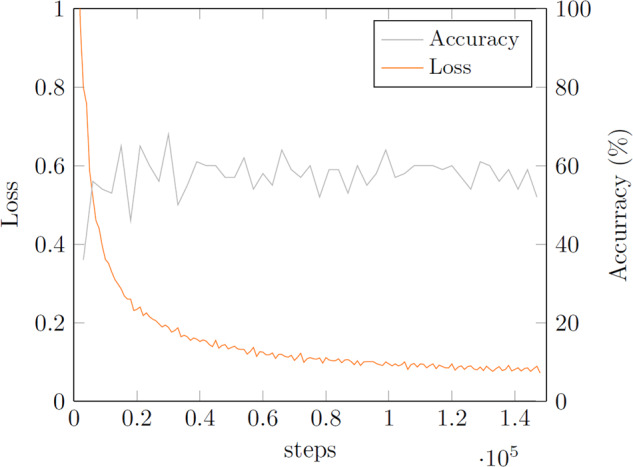
Model 1 training.

To demonstrate the usability of *DOES* and provide a baseline for classification accuracy, a PreActResNet18^30^ was trained over 50 epochs with batch size 32 and class weights on the tiled train data in experiment I. Figure 6 shows accuracy and loss curves for the training (accuracy on test set), and Table 7 provides the confusion matrix on the tiled test set.

**Table 7:** Confusion matrix for model 1 on tiled test set (BG - Background).

As one can see in Fig. 6, the accuracy does not increase significantly anymore after the first 10 epochs (9000 Steps). This indicates training could have been aborted earlier; however, for the sake of a standardized setup, all models were trained over the same number of epochs. Since the best-performing model was kept, attained after epoch 10, not the one after the last epoch, this is not problematic. The confusion matrix in Table 7 shows very well how some categories are more similar to one another while others are more easily discernible. This is at least partially due to the nature of the European scrap grade classification (see also Table 1) and to the way scrap is handled and traded. For example, E6-cubes will sometimes unravel during scrap handling, making them essentially identical to E8. Thus piles of E6 on a scrap yard will usually also contain instances looking like E8-scrap. Also, in scrap trading, batches of e.g. E3 will always also contain individual items that are closer to E1 than E3, and vice versa. If a perspective buyer finds that the scrap was not sorted well enough for the category it is declared as, he might ask for a discount or decline the purchase. Similarly, individual items of E2 scrap can look very much like E3 scrap, e.g. a railroad track that was discarded due to production errors (E2) looks very much like a railroad track discarded after end-of-use (E3). Overall, piles of E2 will have a different average composition than e.g. piles of E3, but there can always be individual items or sections of the pile that cannot be clearly categorized as the one or the other solely from appearance. Thus a 100% accuracy for scrap classification from visual information alone is unrealistic. However, this does not make visual scrap discrimination obsolete. A pile of scrap of one of the categories as a whole will in general display sufficient class characteristics for discrimination, and categories that can more easily be mistaken for one-another also tend to have more similar compositions, thus making a mistake less fatal. It is also visible in the confusion matrix in Table 7 that the model performs better on classes with more examples in the dataset, and, for two classes that are hard to distinguish, tends to pick the more common one such as E8 or E3.

The performance of the PreActResNet18 trained on *DOES* is in a similar range to the published results for PreActResNet on Tiny ImageNet^31,32^. Tiny ImageNet is comparable in size to *DOES*, but features a downsampled version of ImageNet. This shows that *DOES* provides meaningful input for training models and provides a useful alternative as dataset not only for researching on or developing scrap-related solutions, but also to the more general research community interested in a broader approach to computer vision topics than only focusing on the classical “object”-categories.

#### Validation of the tile approach

To validate the usefullness of the tiling approach for dataset generation, experiments II & III were conducted in which a PreActResNet18^30^ was trained over 50 epochs with batch size 32 and class weights on the raw untiled images.

**Fig. 7:**
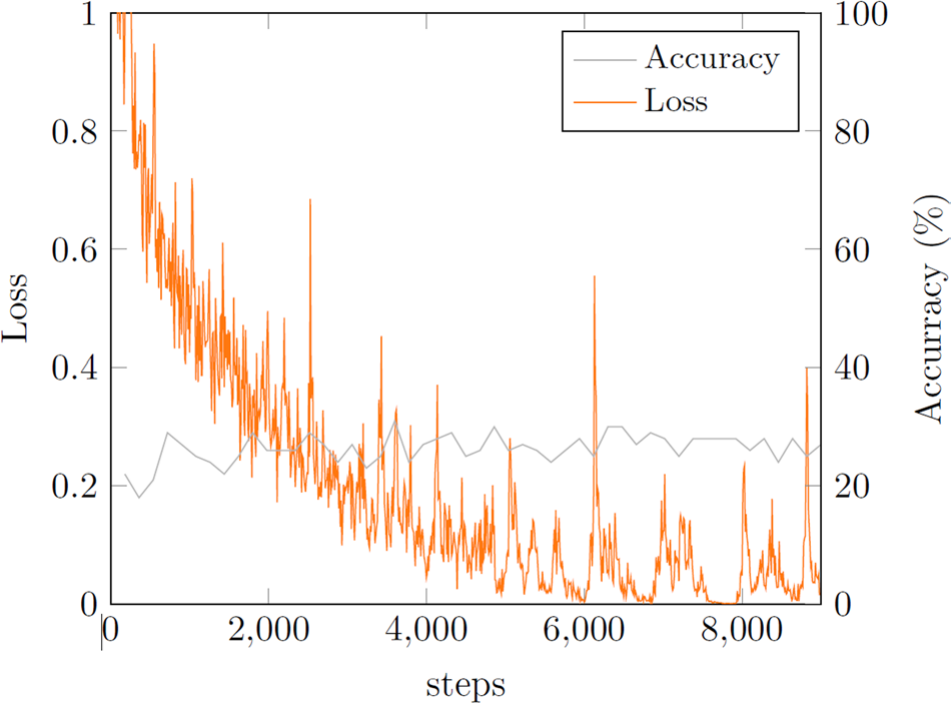
Model 2 training.

As one can see in Fig. 7 and in the confusion matrices in Tables 8, 9, the performance of the model trained on the raw dataset (model 2, experiments II, III) is considerably lower than the model trained on the tiled dataset (model 1 & experiment I, Fig. 6 and Table 7), even when evaluating the model on the raw test set (Table 9), which is structurally more similar to the raw train set then the pre-processed tiled test set. This shows the benefit of the novel approach for dataset generation for “stuff”-like categories. The number of steps in Fig. 7 is lower than e.g. for model 1 in Fig. 6 as the raw dataset is considerably smaller; thus one epoch consists of less steps of 32-image batches than for the tiled dataset. Again, as for the case in experiment I, the accuracy does not increase significantly anymore after the first few epochs (one epoch corresponds to roughly 180 steps), but again, as the best model was saved, attained after epoch 35, not the one after epoch 50, this does not affect the final models performance negatively. The loss curve in Fig. 7 is less smooth than for example for model 1 in Fig. 6. This is because the loss was logged at a more granular scale (in terms of steps) for the smaller raw train set of model 2 as compared to the training instances on the larger tiled train set as for model 1.

**Table 8:** Confusion matrix for experiment II on tiled test set (BG - Background).

**Table 9:** Confusion matrix for experiment III on raw test set (BG - Background).

#### Impact of pretraining

**Fig. 8:**
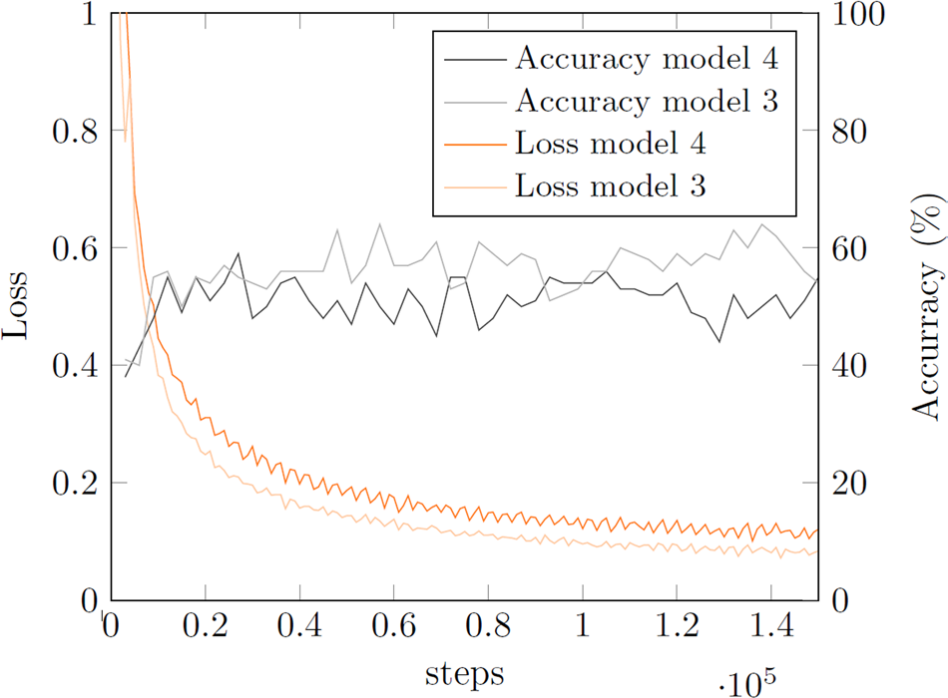
Model 4 on tiled test set and model 3 on tiled test set.

Another interesting aspect is the impact of using a model pretrained on a ‘classical’ object-focused dataset. If *DOES* should be intrinsically similar to ‘classical’ object-focused datasets, pretraining on one of the large available object-focused datasets such as ImageNet should improve model performance. To this end, experiments IV and V were conducted, in which a ResNet50^33^ pretrained on ImageNet and a PreActResNet18 not pretrained on any dataset were trained, both without class weights. If the model 4 in experiment V profited from the pretraining, one would expect the performance to increase as compared to the non-pretrained model 3 in experiment IV. The more so as ResNet50 is a larger model than PreActResNet18, which in itself should encourage better performance. Accuracy and loss during the training process (accuracy on tiled test set) are given in Fig. 8 and the confusion matrices on the tiled test set are provided in Tables 10, 11.

**Table 10:** Confusion matrix for experiment IV on tiled test set (BG - Background).

**Table 11:** Confusion matrix for experiment V on tiled test set (BG - Background).

The results on the test set displayed in Fig. 8 and Tables 10, 11 suggest that the ResNet50 could not profit from pretraining on ImageNet. This could imply that the images in *DOES* are structurally different and the extracted and learned features relevant for discriminating classes are significantly different for *DOES* as compared to the “object”-focused ImageNet dataset. This emphasizes once more the need for providing datasets such as *DOES* for “stuff”-categories, also to enable more research for this kind of data. The loss- and accuracy-curves in Fig. 8 for models 3 and 4 show a very similar progress, and also the confusion matrices in Tables 10, 11 are quite similar, with the model 3 in experiment IV showing slightly better overall results than the model 4 in experiment V. As in the previous experiments, the accuracy does not increase continuously over the entire 50 epochs, but again, as for the previous experiments, the best model was saved, which was attained after epoch 19 for model 3 and after epoch 9 for model 4.

## Data Availability

The dataset is freely available as described in Data Records. The custom code to generate or process these data can be found in the following GitHub repository: https://github.com/micschaefer/does-utils. The rights to the source code of the validation model belong to Saarstahl AG and unfortunately cannot be published.
